# The results of short-course acoustic test could act as an effective predictor of the efficacy of customized music therapy for chronic tinnitus

**DOI:** 10.3389/fnins.2025.1544723

**Published:** 2025-05-14

**Authors:** Tianyi Ni, Yun Jiang, Ziyuan Lin, Jian Ruan, Yulu Wang, Yuehong Liu, Zhao Han

**Affiliations:** ^1^Department of Otorhinolaryngology Head and Neck Surgery, Huadong Hospital Affiliated Fudan University, Shanghai, China; ^2^Geriatric Tinnitus and Deafness Center of Huadong Hospital, Shanghai, China

**Keywords:** short-course acoustic test, customized music therapy, predictor, efficacy, chronic tinnitus

## Abstract

**Objectives:**

The aim of our study is to explore the predictive value of the short-course acoustic test in determining the efficacy of customized music therapy for chronic subjective tinnitus.

**Design:**

Patients with tinnitus as one of the core complaints were included from October 2022 to June 2024. According to the short-course acoustic test results, the participants were divided into three groups: tinnitus disappeared group, tinnitus relieved group and tinnitus unchanged group. All the participants indiscriminately underwent the 10-months of customized music therapy (CMT) and follow-up visits.

**Results:**

Before therapy, only the duration of tinnitus was significantly different among the three groups (*p* < 0.001). After 10-months of CMT treatment, the Tinnitus Loudness Visual Analogue Scale (THI), Hospital Anxiety Scale (HADS-A) and Tinnitus Loudness Visual Analogue Scale (VAS) scores of the three groups showed a decreasing trend (*p* < 0.05), and the treatment efficacy rate of the tinnitus disappeared group was higher than that of the tinnitus relieved group, while the treatment efficacy rate of the tinnitus relieved group was higher than that of the tinnitus unchanged group. The logistic regression results showed that the results of the short-course acoustic test, baseline THI scores, and the presence of hearing loss had significant effects on the efficacy of CMT.

**Conclusion:**

The short-course acoustic test to some extent can predict the efficacy of CMT in patients with chronic subjective tinnitus and can be used to guide clinical therapy.

## 1 Introduction

Tinnitus is defined as the conscious perception of meaningless sounds in the absence of external auditory stimulation (Ghodratitoostani et al., 2016). Approximately 14.4% of adults have a prevalence of tinnitus (Jarach et al., 2022), which often accompany symptoms including depression, anxiety and insomnia, affecting quality of life (Zöger et al., 2006). The more widely accepted mechanism for tinnitus production is the result of plasticity changes in the cerebral cortex following a reduction in peripheral auditory afferents (Rauschecker et al., 2010).

Tinnitus cannot be attributed to identifiable medical causes in most cases, and therefore curative treatments remain unavailable (Cima et al., 2019). The American Academy of Otolaryngology has suggested sound therapy as a potential option for the management of persistent and bothersome tinnitus when patients are counseled to maintain expectations of potential treatment benefits, although the evidence supporting its efficacy remained limited due to high risk of bias and small sample sizes (Tunkel et al., 2014). Based on reversible cortical plastic change, sound therapy can compensate for the lack of peripheral afferents and reverse this adverse remodeling (Pantev et al., 2012). In terms of the presence of sound effect, sound therapy also comes in many other principles, including masking, residual inhibition, lateral inhibitory and desynchronization (Searchfield, 2021). We explored an intensive and regular customized music therapy (CMT) combined with a follow-up and counseling system for patients with chronic subjective tinnitus, targeting the tinnitus frequency range. We previously have demonstrated considerable efficacy using the treatment, probably because central plastic changes were reversed in patients with tinnitus (Liu et al., 2023).

The personalized treatment in CMT was accomplished through a user-input driven artificial intelligence algorithm called “simulated tinnitus brain.” The algorithm integrated the user-input information, including the audiogram, scale scores, tinnitus side, psychoacoustic measurement of tinnitus, combined with the patient’s subjective perception of tinnitus sound, to characterize the patient’s tinnitus percept then develop a sound profile that would theoretically generate and prolong residual inhibition (RI), and therefore suppress the tinnitus sound. Given that CMT is a treatment that requires long-term adherence, we should investigate whether we can find a short-course validated method to predict the potential efficacy of CMT. While a recent study has revealed treatment duration and baseline Tinnitus Handicap Inventory score could predict the efficacy of sound therapy (Gu et al., 2024), an acoustics-based predictive paradigm could reflect the neurophysiological mechanisms underlying tinnitus generation and maintenance. Tinnitus multielement integration sound therapy (T-MIST), a personalized sound that integrated psychoacoustic measurement of tinnitus, hearing level and the subjective preference for background music, was adopted as a short-course acoustic test in this study which relies on the principle of complete masking sound to produce temporary RI (Yan et al., 2021). RI refers to the short-term suppression of tinnitus perception following exposure to an external acoustic stimulus (Schoisswohl et al., 2021). Clinical studies indicated that the phenomenon was displayed in 88% tinnitus patients, though the duration and depth of suppression exhibited significant interindividual variability, and patients would experience more prolonged and robust RI after long-term sound training designed to induce RI (Vernon and Meikle, 2003). Therefore, the aim of our study was to explore the predictive value of the short-course acoustic test in determining the efficacy of CMT for chronic subjective tinnitus.

## 2 Materials and methods

### 2.1 Participants

Patients with the primary complaint tinnitus who presented to the ENT outpatient department from October 2022 to June 2024 were included in our study. Inclusion criteria: Tinnitus patients with age ≥18 years, duration of tinnitus ≥6 months, unilateral or bilateral subjective tinnitus, pitch-matchable tinnitus, normal external and middle ear function, and with the ability to complete the internet-based CMT. Exclusion criteria: Tinnitus patients with severe sensorineural hearing loss (pure-tone hearing threshold >60 dB HL) and organic ear pathology, including ear canal and middle ear tumors, acoustic neuroma and otitis media (underwent MRI to rule out if necessary), pulsatile tinnitus, including myoclonus and vascular tinnitus, Menière’s disease, pregnancy or systemic diseases that could lead to tinnitus, including immune diseases, hypertension and hyperthyroidism. All participants had tried various treatments, including drugs and tinnitus retraining therapy (TRT), a standardized protocol combining directive counseling and sound therapy (Jastreboff, 2007), but in vain.

All patients were given informed consent before the trial. This study is in accordance with the Declaration of Helsinki and was approved by the institutional review board of Huadong Hospital affiliated to Fudan University, China (NO. 2023 K173).

### 2.2 Tinnitus comprehensive evaluation

Tinnitus comprehensive evaluation methods relied on audiological, psychoacoustic measurements and self-report questionnaires. Audiological and psychoacoustic characteristics were measured on the tinnitus sound therapy pairing platform (SFTest330, developed by Foshan BozyTM Medical Technology Company and corresponded to GB/T 16296.2–2016 standards) using the SoCoding wireless encoded headphones and a dedicated high-precision audio processor (Model: 1616) with a frequency response range of 125 Hz–16 kHz and a total harmonic distortion <0.1% by the same experienced audiologist in the soundproof room. Full precision hearing measurement comprised conventional pure-tone audiometry (125 Hz, 250 Hz, 500 Hz, 1 kHz, 2 kHz, 4 kHz, 8 kHz) and extended high-frequency audiometry (10 kHz, 12.5 kHz, 16 kHz), to seek for “hidden hearing loss.” The 3-down, 1-up procedure was used: The stimulus was presented three times, and when turning the loudness up, the stimulus was presented once. Patients were asked about “Was it this one?” and were instructed to raise their right hands when they heard any sound, no matter how small, even if the sound was very faint. The average pure-tone hearing threshold (PTA) at 500, 1000, 2000, and 4,000 Hz were calculated. When the PTA was less than 25 dB HL, they were considered to have no hearing loss.

The tinnitus frequency in Hz and tinnitus loudness in dB SL (sensation level) were estimated via psychoacoustic measurement. The first step of tinnitus frequency matching was to provide a pure tone at 10 dB above threshold in both 0.5 and 1 octave steps from 125 Hz to 16 kHz, alternately distinguishing to match the personalized tinnitus frequency. The duration of each tone was 1 s, and the procedure was repeated a minimum of three times to ensure consistency. Patients were instructed to raise their right hands to report the frequency that gave the closest match to their tinnitus pitch. If patients failed to match the tinnitus frequency, we incorporated at most 1/24-octave pure-tone or the other sound type (narrowband noise, white noise) within the frequency range of the tinnitus pitch. If a match with tones was not found then the patient was excluded in the study. After the completion of tinnitus frequency matching, loudness matching was performed using a 3-down, 1-up procedure: When turning the loudness down, the stimulus was presented three times, and when turning the loudness up, the stimulus was presented once. Patients were asked about “Was it this one?” and were instructed to raise their right hands if the presented stimulus matched their tinnitus loudness. The procedure was performed in the similar manner in the audiological measurement. The loudness matching started at 10 dB above the threshold and the level was progressively decreased or increased in 1 dB step until the tone matched their tinnitus loudness (Henry, 2016). The total process was repeated a minimum of three times for each patient. Patients with bilateral tinnitus were instructed to match the frequency to both sides separately and select the side the tinnitus percept was greatest for our study.

In addition, we assessed the severity of tinnitus with the Tinnitus Handicap Inventory (THI), the subjective loudness of tinnitus with the Tinnitus Loudness Visual Analogue Scale (VAS), and concomitant symptoms including anxiety and depression with the Hospital Anxiety and Depression Scale (HADS). THI was used as the main indicator of CMT efficacy, at pre-therapy and 10-months post-therapy. The THI is the most widely used self-report instrument among researchers and consists of 25 items assessing the patient’s perception of tinnitus and impacts of tinnitus on daily life on three different dimensions (functional, emotional and catastrophic). The total score ranges from 0 to 100 depending on the degree to which the patient is negatively affected by tinnitus. The negative impact of tinnitus on life was positively correlated with the total score. The loudness VAS assesses the subjective intensity of tinnitus, ranging 0 (no tinnitus perceived) to 10 (loudest imaginable tinnitus) (Happich and von Lengerke, 2005). The higher the score, the louder the tinnitus. HADS consists of two subscales, anxiety (HADS-A) and depression (HADS-D). Depending on the severity of anxiety or depressive symptoms, the subscales range from 0 to 21, with a score of 0–7 representing no symptoms and a score of 8–21 representing possible pathological or clinical symptoms (Andersson et al., 2003).

### 2.3 Short-course acoustic test

By means of T-MIST (Tinnitus Multielement Integration Sound Therapy), short-course acoustic test was performed on the tinnitus sound therapy pairing platform (SFTest330, developed by Foshan BozyTM Medical Technology Company) using the SoCoding wireless encoded headphones described above. T-MIST components include three: I-tone, Transfocus and D-sound (Zhao et al., 2019). I-tone was a narrow-band noise with a bandwidth of 1/6 octave, which was used to finely match the tinnitus frequency and activate the hair cells in a specific area to saturate the discharge rate (Zhao et al., 2019; Lu et al., 2019). The sound level of I-tone was set to match or moderately surpass the tinnitus loudness level measured before. Transfocus is the music designed to focus on psychological perception and has nothing to do with frequency, with the spectrum typically covering 125 Hz to 8,000 Hz. D-sound is the sound with natural environment as the background, which has both frequency and psychological characteristics, with the spectrum concentrated between 500 Hz and 5,000 Hz, depending on low/mid/high tinnitus frequency. The sound levels of Transfocus and D-sound in the mixture were adjusted to match the hearing levels. The sound enrichment increased the related neuronal activities between auditory cortex and cognitive-emotional networks (Zhao et al., 2019). The maximum loudness could generally not exceed the patient’s tinnitus loudness matching level by 10 dB SL. For unilateral tinnitus, binaurally presented compound sound was generated based on the psychoacoustic parameters extracted from the affected ear. Dichotic stimulation was employed for bilateral tinnitus, with ear-specific compound sound delivered to each ear according to its respective tinnitus characteristics. Based on the principle of complete masking sound, T-MIST could mask or cover up the tinnitus and reduce the activity level of tinnitus-related neurons to improve tinnitus symptoms. Patients were instructed to focus on compound sound and to ignore tinnitus using the SoCoding wireless encoded headphones. The duration of T-MIST was 15 min. According to the subjective feelings of patients via the VAS score before and immediately after acoustic test, the participants were divided into three groups: tinnitus disappeared, tinnitus relieved, and tinnitus unchanged (Liang et al., 2022). Specifically, if the VAS score decreased to 0 shortly after the test, the patient was assigned to the tinnitus disappeared group; if the VAS score showed a noticeable reduction but did not reach 0, the patient was assigned to the tinnitus relieved group. If the tinnitus VAS score remained unchanged, the patient was assigned to the tinnitus unchanged group (Yan et al., 2021).

### 2.4 Interpretative consultation and customized music therapy (CMT)

Before CMT, all participants were provided with face-to-face standardized interpretative conversation and education via the standardized PowerPoint presentation. The principal contents included a description of: the structure of the auditory system, the mechanism of tinnitus (compensatory response to deafferentation of auditory system, hyperactivity and hypersynchrony), tinnitus neurophysiological model and tinnitus-related distress such as anxiety, depression, and insomnia. Our CMT concentrated not only on the auditory system, but also on the autonomic nervous system and the limbic emotional system. Before CMT, all patients were informed that they would be randomly provided with one of the two interventions: the regular CMT or the placebo CMT. Actually, the regular CMT was provided for all patients other than the placebo CMT to avoid potential placebo effects.

Depending on the Tinnitus Assistant Application (developed by Suzhou Sound Oceans CO., LTD), all patients are provided with CMT, which is individually customized according to the user-input personalized information, including the audiogram, scale scores, tinnitus side, psychoacoustic measurement of tinnitus and the patient’s subjective perception of tinnitus sound. The core principle of the application is the simulated tinnitus brain as a user-input driven artificial intelligence algorithm to integrate patient’s information to characterize the patient’s tinnitus percept then develop a sound profile that would suppress the tinnitus sound. The first step of music production is to select the type of tinnitus sound based on the patient’s subjective perception from normal daily life sounds, electronic sounds and common tinnitus sounds. The next step is to select further categories. For example, normal daily life sounds comprise kettle boiling sounds and television snowflake (resembling the hissing or buzzing sound of an analog television with no signal); electronic sounds include mechanical sounds and motor sounds; common tinnitus sounds include tonal and ringing sounds. The third step is to measure the tinnitus frequency and loudness. The fourth step is to locate the overactive brain areas associated with tinnitus using the built-in simulated tinnitus brain. The music is then customized through an anti-phase editing program with the same intensity and frequency as the tinnitus sound and 180 degrees out of phase, eliminating the tinnitus by theoretically reducing cortical perception. For unilateral tinnitus, binaurally presented customized music was generated based on the psychoacoustic parameters extracted from the affected ear. Dichotic stimulation was employed for bilateral tinnitus, with ear-specific customized music delivered to each ear according to its respective tinnitus characteristics.

Patients are instructed on the usage of the EdifierW800BT headphones and maintain the music loudness slightly lower than the tinnitus loudness to ensure that the patients can hear both the tinnitus and the music throughout. In addition, they were instructed to complete CMT in a quiet environment for over 30 min each time and for a total duration of over 2 hours per day.

### 2.5 Follow-up and counseling system

**Figure 1:**
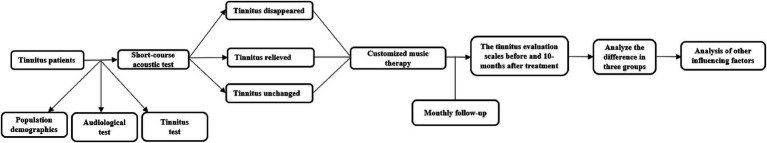
The flow diagram of this study.

All participants underwent 10-months of CMT and completed the follow-up assessments during daytime hours (8:00 AM to 6:00 PM) at monthly intervals of 30 ± 3 days from the baseline visit via the Tinnitus Assistant Application. The follow-up and counseling system could record the therapy time and supervise the patients to complete their music therapy daily and follow-up questionnaires monthly with the aid of artificial intelligence. The monthly follow-up questionnaires, including THI, VAS and HADS, were required to be completed on time to allow evaluation of the phased treatment outcome. Patients could consult with the doctor/audiologist about treatment-related problems and receive prompt feedback during working hours. The doctor/audiologist also communicated with patients regularly and provided psychological counseling. Tinnitus matching was performed regularly to avoid an altered tinnitus pitch and to re-customize the music when necessary. The criterion for effective cure was defined as a reduction in tinnitus THI scores of over 20 points after 10-months of therapy (Choy et al., 2010). The flow diagram is shown in Figure 1.

### 2.6 Statistical methods

Statistical analyses were conducted utilizing SPSS statistics software (version 25.0, SPSS Inc., Chicago, IL, USA). Firstly, data were analyzed to test whether there were differences, across the three groups, in: population demographics, tinnitus characteristics, hearing level and THI, VAS, HADS-A and HADS-D scores before treatment via Kruskal-Wallis and Chi-square tests. Secondly, the Wilcoxon signed-rank test was used to compare the THI, VAS, HADS-A, HADS-D scores before and after 10-months of CMT in the three groups of patients, respectively. Thirdly, the treatment efficacy among the three groups was compared via the Chi-square test. Lastly, multivariable logistic regression, a form of generalized linear model, was conducted to explore the independent factors affecting the treatment efficacy. The model simultaneously included all independent variables to evaluate their independent effects while controlling for potential confounders. The outcome variable was the long-term treatment efficacy, which was dichotomized as effective cure (the reduced THI ≥ 20 points) or ineffective cure (the reduced THI < 20 points). The independent variables included both continuous and categorical variables. The continuous variables were age, tinnitus frequency, tinnitus duration, the baseline THI, VAS, HADS-A and HADS-D scores. The categorical variables included the results of the short-course acoustic test, gender, and tinnitus side. *p* < 0.05 represented statistical significance.

## 3 Results

### 3.1 Participants characteristics

The study included 515 patients who met the criteria (see Table 1 for details). We classified patients as tinnitus disappeared group (*n* = 80, accounting for 16% of the total number), tinnitus relieved group (*n* = 228, accounting for 44% of the total number) and tinnitus unchanged group (*n* = 207, accounting for 40% of the total number) based on the short-course acoustic test. The tinnitus disappeared group consisted of 44 males and 36 females, with the median and interquartile range (25–75th) age of 49 (41–57) years and tinnitus duration of 1 (1-2) years. The tinnitus relieved group consisted of 122 males and 106 females, with the median and interquartile range (25–75th) age of 51 (44–58) years and tinnitus duration of 1 (1-5) years. The tinnitus unchanged group consisted of 103 males and 104 females, with the median and interquartile range (25–75th) age of 50 (42–57) years and tinnitus duration of 2 (1-7) years.

**Table 1:** Baseline characteristics of the patients in three groups (*N* = 515).

Patient group	Tinnitus disappeared	Tinnitus relieved	Tinnitus unchanged	*p*
Number	80	228	207	
Gender (male/female)	44/36	122/106	103/104	0.637
Age (year)	49 (41–57)	51 (44–58)	50 (42–57)	0.563
Tinnitus side (left/right/both)	20/16/44	66/38/124	64/31/112	0.810
Tinnitus duration (year)	1 (1–2)	1 (1–5)	2 (1–7)	<0.001
THIB	63 (37–82)	52 (36–74)	56 (38–76)	0.136
VASB	5 (4–8)	6 (4–8)	6 (4–8)	0.183
HADS-AB	10 (7–12)	9 (7–12)	9 (7–11)	0.416
HADS-DB	7 (4–10)	7 (4–10)	7 (4–10)	0.804
Tinnitus frequency (Hz)	7,000 (4000–10,000)	6,000 (4000–9,000)	5,000 (4000–9,000)	0.260
PTA (dB)	21 (14–32)	24 (16–36)	25 (16–34)	0.084

The numerical values are presented as median with the interquartile range (25–75th). The categorical values are presented as the number of cases. THIB, VASB, HADS-AB, HADS-DB, THI before treatment; VAS before treatment; HADS-A before treatment, HADS-D before treatment; PTA, pure-tone average hearing thresholds of 500, 1,000, 2000 and 4,000 Hz.

The three groups significantly differed in tinnitus duration (*p* < 0.001) before therapy. After adjusting for multiple comparisons, the only significant difference observed was between the tinnitus disappeared group and the unchanged group (*p* = 0.001). The three groups did not significantly differ in gender, age, tinnitus side, tinnitus frequency, PTA, the maximum hearing loss. Meanwhile, the baseline tinnitus severity (as assessed with THI), tinnitus distress (as assessed with VAS), anxiety status (as assessed with HADS-A) and depression status (as assessed with HADS-D) did not significantly differ in the three groups. The other baseline characteristics for details are shown in Table 1.

### 3.2 Curative efficacy of CMT in three groups

The evaluation for curative effect relied on changes in THI score between pre-therapy and 10-months post-therapy. Table 2 demonstrates that the median THI score decreased from 63 (37–82) to 12 (2-37), the median loudness VAS score decreased from 5 (4-8) to 3 (1-5), the median HADS-A score decreased from 10 (7-12) to 4 (2-7), and the mean HADS-D score decreased from 7 (4-10) to 3 (1-7) in tinnitus disappeared group. There were statistically significant differences in the median THI, VAS, HADS-A and HADS-D score between pre-therapy and 10-months post-therapy (*p* < 0.001).

**Table 2:** Treatment outcomes after 10-months of CMT in three groups.

Group	Scale	Pre-therapy	10 M-post-therapy	*Z*	*p*
Tinnitus disappeared	THI	63 (37–82)	12 (2–37)	−7.711	<0.001
	VAS	5 (4–8)	3 (1–5)	−7.099	<0.001
	HADS-A	10 (7–12)	4 (2–7)	−7.153	<0.001
	HADS-D	7 (4–10)	3 (1–7)	−6.064	<0.001
Tinnitus relieved	THI	52 (36–74)	35 (16–48)	−10.998	<0.001
	VAS	6 (4–8)	4 (3–6)	−9.079	<0.001
	HADS-A	9 (7–12)	7 (4–8)	−8.830	<0.001
	HADS-D	7 (4–10)	4 (1–8)	−5.937	<0.001
Tinnitus unchanged	THI	56 (38–76)	48 (32–66)	−2.822	0.005
	VAS	6 (4–8)	5 (3–7)	−5.187	<0.001
	HADS-A	9 (7–11)	8 (5–10)	−5.678	<0.001
	HADS-D	7 (4–10)	7 (3–9)	−1.592	0.111

The values are presented as median with the interquartile range (25–75th). THI, Tinnitus Handicap Inventory; VAS, Visual Analogue Scale; HADS, Hospital Anxiety and Depression Scale; A, Anxiety; D, Depression.

The median THI score decreased from 52 (36–74) to 35 (16–48), the median loudness VAS score decreased from 6 (4-8) to 4 (3-6), the median HADS-A score decreased from 9 (7-12) to 7 (4-8), and the mean HADS-D score decreased from 7 (4-10) to 4 (1-8) in tinnitus relieved group. Statistically significant differences were observed in the median THI, VAS, HADS-A and HADS-D score between the values obtained at pre-therapy and post-therapy time points (*p* < 0.001).

The median THI score decreased from 56 (38–76) to 48 (32–66), the median loudness VAS score decreased from 6 (4-8) to 5 (3-7), the median HADS-A score decreased from 9 (7-11) to 8 (5-10), and the mean HADS-D score decreased from 7 (4-10) to 7 (3-9) in tinnitus unchanged group. There were statistically significant differences in the median THI (*p* = 0.005), VAS (*p* < 0.001) and HADS-A (*p* < 0.001) score between pre-therapy and 10-months post-therapy, but not in the median HADS-D score (*p* = 0.111).

### 3.3 Comparison of effective treatment among three groups after 10-months of CMT

As mentioned above, the criterion for effective treatment is a reduction in tinnitus THI score of 20 points or more after 10-months of therapy. According to this criterion, after 10-months of CMT treatment, tinnitus severity was effectively treated in 73.8% of patients in the tinnitus disappeared group, 43.4% in the tinnitus relieved group, and 22.2% in the tinnitus unchanged group. The difference in effective cure rates after treatment among the three groups was significant (*p* < 0.001). After adjustment for multiple comparisons, the proportion of effective treatment was significantly higher in the tinnitus disappeared group than in the tinnitus relieved group, while the proportion of effective treatment was significantly higher in the tinnitus relieved group than in the tinnitus unchanged group. The details are shown in Table 3.

**Table 3:** Proportion of the effective treatment between the three groups of patients.

Item	Tinnitus disappeared	Tinnitus relieved	Tinnitus unchanged	*p*
Effective treatment	59 (73.8%)	99 (43.4%)	46 (22.2%)	<0.001
Ineffective treatment	21 (26.2%)	129 (56.6%)	161 (77.8%)	

Effective treatment, THI score drops 20 or more.

### 3.4 Binary logistic regression of the factors affecting the effective treatment after 10-months of CMT

As is shown in Table 4, the side, duration and frequency of tinnitus, gender, age, the baseline VAS, HADS-A and HADS-D scores exert no effect on the effective treatment of CMT (*p* > 0.05). On the contrary, the short-course acoustic test results, the baseline THI scores and PTA significantly influenced the effective treatment. Notably, the effective treatment of the tinnitus disappeared and relieved group was 5.246 times better than of tinnitus unchanged group. The baseline THI scores were positively associated with the effective treatment (*p* < 0.001; OR = 1.056, 95% CI = 1.039–1.073). Each additional point of the baseline THI scores increased the incidence of long-term effective treatment by 5.6%. PTA was found to have a significant negative association with the effective treatment (*p* = 0.003; OR = 0.978, 95% CI = 0.963–0.993). For every dB increase in PTA, the incidence of long-term effective treatment decreased by 2.3%.

**Table 4:** Analysis of factors affecting the effective treatment after 10 months of CMT.

Influencing factors	*B*	SE	Wald	*p*	OR	95% CI
Short-time acoustic test (Tinnitus disappeared and relieved vs. Tinnitus unchanged)	1.657	0.241	47.351	<0.001	5.246	3.272–8.410
THI scores (per point)	0.054	0.008	45.464	<0.001	1.056	1.039–1.073
Age (per year)	0.003	0.010	0.094	0.760	1.003	0.983–1.023
Gender (male vs. female)	−0.077	0.220	0.124	0.725	0.926	0.602–1.423
Tinnitus frequency (per Hz)	0.000	0.000	0.234	0.629	1.000	1.000–1.000
Tinnitus duration (per year)	−0.016	0.016	1.065	0.302	0.984	0.954–1.015
Tinnitus side (unilateral vs. bilateral)	−0.212	0.221	0.922	0.337	0.809	0.524–1.247
PTA (per dB)	−0.022	0.008	8.563	0.003	0.978	0.963–0.993
HADS-A scores (per point)	−0.024	0.039	0.392	0.531	0.976	0.905–1.053
HADS-A scores (per point)	0.029	0.033	0.752	0.386	1.029	0.964–1.099
VAS scores (per point)	−0.045	0.062	0.522	0.470	0.956	0.847–1.080

Effective treatment, THI score drops 20 or more. THI, Tinnitus Handicap Inventory; VAS, Visual Analogue Scale; PTA, pure-tone average hearing thresholds of 500, 1,000, 2000 and 4,000 Hz; HADS, Hospital Anxiety and Depression Scale; A, Anxiety; D, Depression.

## 4 Discussion

Tinnitus is primarily caused by hearing loss, with its onset linked to plasticity changes in the auditory cortex following decreased peripheral auditory input due to damage to the peripheral auditory system (Knipper et al., 2010). The deprivation of auditory input disrupts the balance of excitation and inhibition in central auditory pathways, leading to increased spontaneous and synchronized discharges in the auditory central neurons (Shore et al., 2016). These alterations often coincide with dysregulation in the autonomic nervous and limbic emotional system (Ghodratitoostani et al., 2016).

Ever since sound therapy for tinnitus was proposed, substantial progress has been made. Neuromonics Tinnitus Treatment combined spectrally modified music with structured counseling, showing significant improvements in tinnitus distress (Davis et al., 2007). Okamoto demonstrated the long-term efficacy of tailor-made notched music in alleviating tinnitus perception by reducing cortical hyperactivity (Okamoto et al., 2010). Acoustic coordinated reset used patterned sound sequences corresponding to the dominant tinnitus frequency to desynchronize pathological neural oscillations and relieved tinnitus symptoms, contributing to long-term and cumulative desynchronizing effects (Adamchic et al., 2012).

The neurophysiological model of tinnitus identified multiple targets of dysfunction, including the peripheral auditory system, auditory and non-auditory cortices, subcortical auditory pathways, the limbic system, and the autonomic nervous system (Jastreboff, 1990). The main challenge in tinnitus treatment was to accurately targeting these areas. The measurable tinnitus frequency could serve as potential targets for music therapy or sound therapy. The complexity of the tinnitus mechanism necessitated the development of personalized CMT tailored to each individual’s unique tinnitus condition. Meanwhile, the combination of music therapy with counseling appeared particularly increasingly promising (Chen et al., 2021). Luo et al. (2025) studied music combined with cognitive behavioral therapy by running a single-arm study, found a significant reduction in scale score, including THI and VAS scores, and enhanced functional connectivity within the frontal–parietal–temporal network. In this study, we employed a web-based CMT with psychological counseling, which used a user-input driven artificial intelligence algorithm to characterize the patient’s tinnitus percept then develop a sound profile that would suppress the tinnitus sound (Liu et al., 2023). The music therapy was designed to predict changes within the tinnitus frequency range, aiming to provide a targeted therapeutic effect, while psychological counseling addressed distress maintenance. Our approach shared conceptual ground with the progressive tinnitus management framework (Edmonds et al., 2017), although the emphasis on sound therapy was different.

A comprehensive follow-up system was implemented to alleviate anxiety, depression and pressure in tinnitus patients. Our previous research has shown that sound therapy or music therapy alone was often insufficient for outpatient tinnitus treatment in the absence of the effective follow-up and psychological intervention, but when combined with the effective follow-up system, outcomes are significantly improved (Wei et al., 2017). While previous small-sample studies have conducted outpatient or telephone follow-up, it seemed impractical for our large-sample study. To address the matter, we developed a web-based follow-up system to ensure consistent and efficient patient monitoring (Liu et al., 2023). However, during the onset of our study, we encountered challenges in maintaining a placebo control group. Patients assigned to the placebo group exhibited high dropout rates and frequently requested to be transferred to the CMT group. Consequently, after careful consideration, all patients were orally informed that they would receive either CMT or placebo music, but in practice, all patients received CMT. Undoubtedly, the procedure made it difficult to distinguish whether our CMT only acts as a placebo. Nevertheless, a previous study by Tong et al. demonstrated that music therapy was superior to tinnitus retraining therapy for alleviating emotional disturbances and reducing tinnitus loudness in chronic subjective tinnitus (Tong et al., 2023). The finding supported our belief that the treatment effect observed in our study was greater than the prediction for a placebo effect.

It is remarkable that the significant reduction in the THI scores in the tinnitus disappeared group reached approximately 50 points, suggesting a robust therapeutic benefit. The reduction was notably higher than the average THI score changes of 10 points in the other two groups. The huge change supported our belief that the short-time acoustic test could predict the efficacy of CMT in chronic subjective tinnitus. Tinnitus progression involves peripheral nervous system damage, abnormal central auditory discharges, subsequent central remolding, and eventual impacts on sleep and emotional states (Simões et al., 2021). Early intervention prior to central remolding typically yields better outcomes, as these changes are more reversible at earlier stages. However, if the disease progresses to or beyond the central remolding stage, treatment becomes extremely challenging (Kleinjung et al., 2024). Our study revealed that patients in the “tinnitus disappeared” group had significantly shorter durations of tinnitus than those in the “tinnitus unchanged” group which suggested that shorter durations of auditory deafferentation allow the central auditory system to remain in the phase of abnormal spontaneous discharges and synchronization, where interventions like T-MIST the narrow-band noise component compensates for the reduction of peripheral afferents by excitation of the residual functional hair cells and suppresses aberrant neuronal activity to achieve temporary elimination of tinnitus. Conversely, in “tinnitus unchanged” group with longer durations of tinnitus, because of the longer peripheral deafferentation time, cortical remodeling—such as synaptic alterations or disrupted network connectivity—rendered tinnitus unchanged after short-course acoustic test. The phenomenon underscored the difference in treatment outcomes between acute and chronic tinnitus. Acute tinnitus often required interventions to correct abnormal synchronization and restore cortical gating, whereas chronic cases involved structural neuronal changes that necessitate prolonged therapy. Indeed, our results indicated that patients who did not respond to short-course T-MIST showed improvements after 10-months of CMT.

The principles of T-MIST involve complete masking to induce residual inhibition (RI), which is a critical concept in acoustic therapy for tinnitus. Masking sounds matched to the tinnitus frequency and intensity could suppress abnormal neuronal discharges in the auditory cortex, thereby alleviating tinnitus (Roberts et al., 2015). However, the average RI duration is limited to approximately 5.1 min (Goldstein et al., 2005), and repeated masking may lead to reduced patient tolerance, hearing loss, or dependency. In contrast, CMT is based on the “simulated tinnitus brain” and the anti-phase editing, which provides patients with sound waves shifted 180 degrees from the tinnitus wave with the aim of generating and prolonging RI. From this point of view, there is a similarity between the two mechanisms. Sendesen and Turkyilmaz (2024a) studied sound treatment by running a controlled study, found sound treatment demonstrated enhanced therapeutic outcomes for patients exhibiting both residual inhibition and frequency-matched hearing loss ranging from 45 to 55 dB HL. The recent study revealed RI could serve as a prognostic indicator for tinnitus sound enrichment treatment used to suppress spontaneous neuronal activity of the central auditory system, with RI-positive patients demonstrating a greater probability of treatment success (Sendesen and Turkyilmaz, 2024b). Although our methodology employed customized music rather than sound enrichment, the common dependence on neural plasticity pathways implied the comparable and operative predictive mechanisms. Notably, the tinnitus disappeared group in our study could represent patients with particularly strong RI capacity, consistent with the RI-positive described by Sendesen and Turkyilmaz (2024b).

Furthermore, both T-MIST and CMT shared a mechanism of compensating for hearing loss by targeting the tinnitus frequency within the range of hearing loss in most cases, also explaining why T-MIST could be used as a predictor of long-term efficacy of CMT. All these could be reflected by the results from our trial, in which a large majority of the patients with positive results of short-term acoustic test had a positive response to CMT, while only a minority of patients had no effect, which could not exclude the possibility that they failed to complete the required duration and frequency of CMT. From the perspective, the implementation of T-MIST and CMT tailored to individual clinical profiles will enable clinicians to make more informed treatment recommendations, potentially representing a step toward addressing tinnitus heterogeneity through personalized medicine.

Despite the widespread acceptance of central reorganization as a key mechanism underlying tinnitus, its etiology remains incompletely understood (Galazyuk et al., 2012). For patients unresponsive to both T-MIST and CMT, nonadherence to prescribed protocols may contribute to poor outcomes. Alternatively, their tinnitus may stem from mechanisms unrelated to frequency-specific hearing loss, as evidenced by cases of tinnitus in individuals with normal hearing thresholds (Han et al., 2021).

This study has several limitations. First, it was not conducted as a standard double-blind randomized controlled trial, which would have been ideal for minimizing bias and placebo effects. However, the design was challenging to implement in the current clinical context, both from a practical, methodological and ethical standpoint. Additionally, the absence of a placebo control group made it difficult to quantify how much the efficacy is attributable to the placebo effect. To address the matter, we will constantly explore methods to implement a more robust follow-up system that allow for the inclusion of a placebo or control group without compromising patient retention and adherence. Future studies should aim to incorporate a control group, using a sham or alternative sound therapy, to better isolate the specific effects of CMT. Second, we did not stratify patients by the degree of hearing loss, although its impact on short-term acoustic therapy and CMT efficacy was considered. Besides, all tinnitus patients included in the study came from China, but not America, Africa and Europe. Therefore, it is necessary to conduct future studies in more diverse populations. Future research should address these limitations to refine treatment strategies and expand our understanding of tinnitus pathophysiology.

## 5 Conclusion

To sum up, our study indicated that it is feasible to reduce tinnitus severity and concomitant symptoms of chronic subjective tinnitus patients by means of an intensive and regular CMT, regardless of the short-time acoustic test results. We recommend the short-time acoustic test results as a potential predictor for the efficacy of CMT in chronic tinnitus.

## Data Availability

The original contributions presented in the study are included in the article, further inquiries can be directed to the corresponding authors.
